# Dataset on soil carbon dioxide fluxes from an incubation with tropical peat from three different land-uses in Jambi Sumatra Indonesia

**DOI:** 10.1016/j.dib.2021.107597

**Published:** 2021-11-19

**Authors:** Louis-Pierre Comeau, Kristell Hergoualc'h, Louis V. Verchot

**Affiliations:** a Fredericton Research and Development Centre, Agriculture and Agri-Food Canada, Fredericton, NB, Canada; b Center for International Forestry Research (CIFOR), Lima, Peru; c The Alliance of Bioversity International and CIAT, The Americas HubKm 17 Recta Cali-Palmira Zip Code 763537 A.A. 6713, Cali, Colombia

**Keywords:** Tropical peat, Land-uses, Incubation, Carbon dioxide emissions, Peat density fractionation, Soil moisture content

## Abstract

Conversion of tropical peat swamp forests to increase and agricultural production has generated substantial peat carbon loss in the Asia-Pacific region. Different land-uses and management practices oxidize the tropical peat at diverse rates due mainly to different water table levels. In recent years, several studies have measured soil carbon dioxide emissions in-situ; however, only few studies have evaluated the effect of moisture on carbon dioxide fluxes in incubation experiments. Here, we present the dataset of an incubation performed with 360 intact peat cores from three different land-uses (i.e. 120 from intact peat swamp forest; 120 from drained logged peat forest; and 120 from oil palm plantation) collected on the peat dome of Jambi Sumatra Indonesia. Different moisture levels in the intact cores were set by either drying the intact peat cores for short period of time or by adding extra water before the incubation. Dynamic dark aerobic incubation in airtight containers coupled with carbon dioxide measurement with an infrared gas analyser and the gas fluxes was used to measure to gas fluxes. The average carbon dioxide fluxes were 5.38 ± 0.91, 4.15 ± 0.35 and 1.55 ± 0.13 µg CO_2_-C g^−1^ h^−1^ for the intact peat swamp forest, drained logged peat forest and oil palm plantation, respectively.

## Specifications Table

**Table:** 

Subject	Agricultural science; Environmental science
Specific subject area	Carbon dioxide gases (CO_2_) emissions from aerobic incubation
Type of data	Tables and Figures.
How data were acquired	Dynamic dark aerobic incubation technique with intact soil cores. Briefly, CO_2_ concentration in the containers was assessed at 0, 24, 48 and 72 hours by connecting the containers in closed system with an infrared gas analyser (IRGA) for two seconds. The CO_2_ flux was calculated with a linear regression done for the four time points of the sample that had a R^2^ > 0.98 [1], [2]. Soil pH was determined with H_2_O with a ratio of 1:4. The bulk density of the peat (g of dry weight per cm^−3^) was determined by dividing the dividing the weight of the soil for an intact fix volume of soil. Samples were collected from the top 0-20 cm soil layer for chemical analysis. Soil samples were fractionated into very light fraction plus light fraction (VLF+LF, density less than 1.25 g ml^−1^) and medium weight fraction plus heaviest peat fraction (MWF+HF, density more than 1.25 g ml^−1^) with a dense liquid (NaI). The fractionated materials were dried, finely ground with a ball mill, and subsequently analyzed for total C and N content using a Costech Elemental Combustion System (Costech Analytical 191 Technologies, Inc.) coupled to a Delta V Advantage Mass Spectrometer (Thermo Fisher 192 Scientific Inc.). The soil was classified according to the World Reference Base (IUSS Working Group WRB, 2006).
Data format	Mixed (raw and pre-processed)
Parameters for data collection	The tropical peat was Hemic Histosol (Dystric, Drainic) (IUSS Working Group WRB, 2006) collected at three different locations corresponding to three different land-uses (LUs) on the alluvial peat plain (peat dome) of Jambi Sumatra Indonesia. The three LUs were: an intact peat swamp forest (PF), a drained logged peat forest (DF) and a 7 year-old oil palm plantation (OP)
Description of data collection	Undisturbed soil cores of volume 313 cm^3^ (inner diameter 8.15 cm, height 6 cm) were collected using a stainless steel core soil sampler from the upper part of the soil profile (0–6 cm). In each LU, the intact cores were collected at random positions in a radius of 50 m. A total 120 samples per LU were collected. Soil cores were kept in their stainless steel containers with impermeable bottom lid until moisture adjustment.
Data source location	The three sampling sites in Jambi Sumatra Indonesia were the PF of Berbak National Park (1°27′S, 104°21′E), and the DF and OP of Bakrie Sumatera Plantation of SNP (Sumber-Tama Nusa Pertiwi) (1°39′S, 103°52′E).
Data accessibility	Data are with the article

## Value of the Data

• The data presented here are important because peatlands play an vital role in regulating the climate by the mean of carbon storage and until now limited data are available on carbon cycling in these ecosystems. This set of data will be useful to establish baselines for peat carbon dioxide flux from tropical regions. These data will benefit research on climate change mitigation mechanisms such as REDD+ (reducing emissions from deforestation and forest degradation) and for national greenhouse gas accounting. Specifically, soil carbon stocks and greenhouse gas modeling algorithms require flux values at different peat moisture contents per temperature level.
• The samples were from an intact peat swamp forest, a drained logged peat forest and a 7 year-old oil palm plantation and will be useful for scientists performing meta-analyses that evaluate potential land-use changes on peat carbon stock and dynamic. Overall, this dataset enhance the available information of carbon dioxide flux produced from incubation studies at different moisture levels.
• The data are from different peat properties with an associated density fractionation determination that also benefit scientists policymakers and specialists working on explaining and extrapolating of carbon dioxide flux from peatlands. This experiment was produced at a temperature of 27°C ±2 and with this baseline further development can be produced to generate efflux insights at lower or higher temperature regimes.

## 1 Data Description

**Fig. 1:**
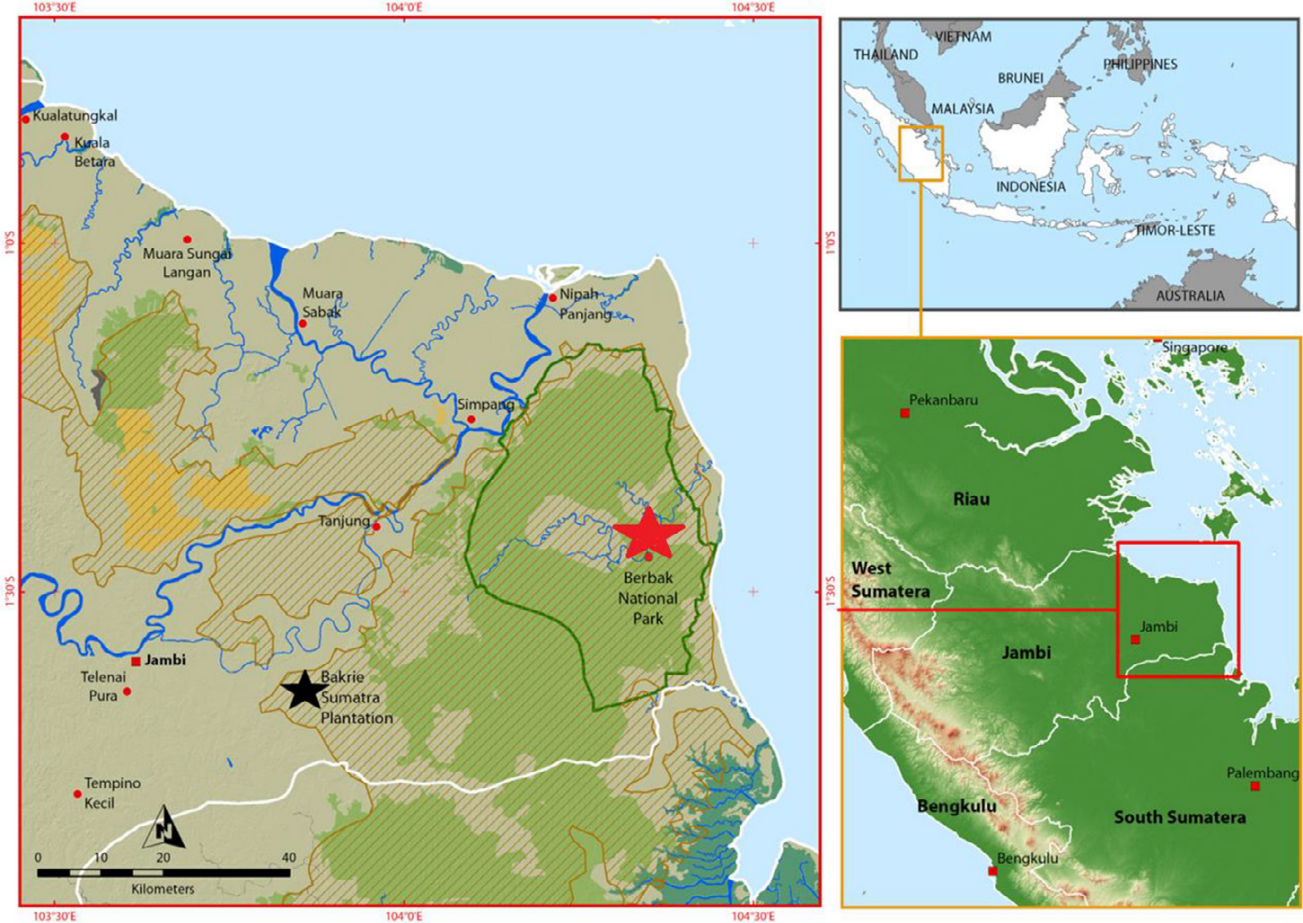
Location of study sites.

**Fig. 2:**
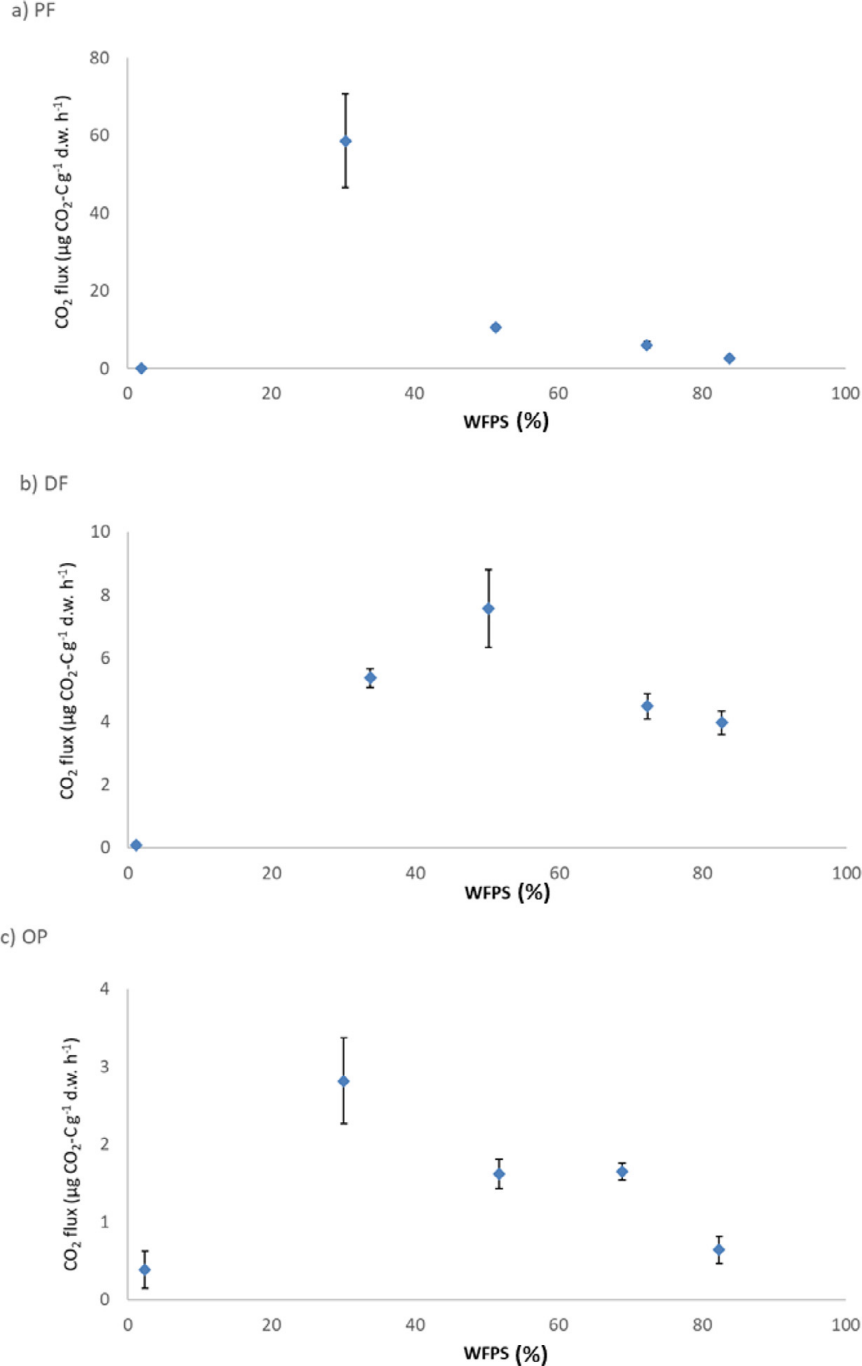
Carbon dioxide fluxes from the incubations: (a) PF, primary peat swamp forest; (b) DF, drained and logged forest on peat soil; (c) 7 year old oil palm plantation on peat soil. WFPS, water filled pore space; d.w., dry weight. Error bars represent the standard error values.

Pristine tropical peat swamp forests conversion productive land-uses has generated substantial peat carbon loss in the Asia-Pacific region [4]. Different land-uses and management practices oxidize the tropical peat at diverse rates due mainly to different water table levels [5]. The present article contains a first figure (Fig. 1) showing the geographical locations where the intact peat cores were collected. That figure displays, on the right, the province of Jambi in Southeast Asia, and on the left, it shows the location of the Berbak National Park (corresponding to the PF site marked with a red star) and the Backrie Sumatra Plantation (corresponding to the DF and OP marked with a black star). The second figure (Fig. 2) contains 3 regression graphs corresponding to intact peat swamp forest (a), drained and logged forest (b) and 7 years old oil palm plantation (c). On these graphs, the “X” axis is the water filled pore space (WFPS) and the “Y” axis is the CO_2_ fluxes with unit of µg CO_2_-C g^−1^ d.w. h^−1^. Table 1. Presents the average CO_2_ fluxes from the incubations at the different water filled pore space (WFPS). From left to right the columns in the table show the three LUs (PF, DF and OP); the WFPS intervals (0–20,20–40, 40–60, 60–80, and 80–100%); the average CO_2_ flux (for each category µg CO_2_-C g^−1^ d.w. h^−1^); the standard error associated with the averages (SE); the samples size for each category (n); and the average WFPS for each category. Table 2. presents the peat soil pH and bulk density at the PF, DF and OP sites, respectively. Table 3. shows the results from the peat density fractionation at the PF, DF and OP site. The density fractions are, very light; light; medium weight; and heavy and for each of them the mass percentage and the C:N ratio is presented. Appendix A, B and C present supporting information as raw data on the 360 intact cores that were used in the incubation (i.e. sample id, moisture pre-handling, CO_2_ flux and water filled pore space) and peat physical and chemical properties.

**Table 1:** Comparison of the average carbon dioxide fluxes from the incubations in the three LU at the different water filled pore space (WFPS).

LU	WFPS interval (%)	Average CO_2_ flux(µg CO_2_-C g^−1^ d.w. h^−1^)	SE	n	Average WFPS in the category (%)
PF	0-20	0.13	0.02	18	1.98
PF	20-40	58.59	11.99	2	30.36
PF	40-60	10.64	0.63	6	51.30
PF	60-80	6.12	0.87	50	72.37
PF	80-100	2.72	0.50	29	83.85
DF	0-20	0.08	0.01	17	1.13
DF	20-40	5.37	0.31	4	33.68
DF	40-60	7.58	1.23	15	50.17
DF	60-80	4.48	0.40	49	72.31
DF	80-100	3.96	0.37	16	82.62
OP	0-20	0.39	0.24	20	2.40
OP	20-40	2.82	0.55	15	30.11
OP	40-60	1.62	0.19	35	51.74
OP	60-80	1.65	0.11	38	68.87
OP	80-100	0.64	0.18	2	82.37

LU, Land-use; WFPS, water filled pore space; SE, standard error; n, sample size; PF, primary peat swamp forest; DF, drained and logged forest; OP on peat soil, 7 years old oil palm plantation on peat soil.

**Table 2:** Average of soil pH and bulk density at the 3 LUs.

LU	Bulk density	pH
	(g d.w. cm^−3^)	(H_2_O 1:4)
PF	0.15 (<0.01)	3.20 (0.02)
DF	0.16 (0.01)	3.28 (0.03)
OP	0.20 (0.01)	3.50 (0.04)

LU, land-use; SE, standard errors.

**Table 3:** Average percentage of C, N and C:N ratio in the top soil peat fractions at the three land-uses (LUs).

	Very light fraction		Light fraction		Medium weight fraction		Heavy fraction	
LU	%	C:N	%	C:N	%	C:N	%	C:N
PF	0.1 (<0.1)	62.3 (17.6)	16.8 (14.0)	45.0 (5.5)	57.6 (16.5)	35.8 (1.2)	12.5 (5.2)	42.0 (3.7)
DF	0.9 (0.5)	36.3 (7.7)	7.9 (3.7)	40.6 (5.3)	33.4 (25.3)	34.0 (3.0)	50.6 (24.8)	34.1 (3.5)
OP	0.7 (0.5)	49.3 (6.2)	15.7 (11.6)	41.8 (2.3)	13.4 (5.0)	38.0 (1.2)	57.7 (16.5)	38.7 (1.3)

^a^ PF, intact peat swamp forest; DF, drained logged forest; OP, 7-year-old oil palm plantation.

^b^ Numbers are means followed by standard errors.

Average %C for all the LU and depths = 55.0 (±0.6)

## 2 Experimental Design, Materials and Methods

### 2.1 Sampling sites

This dataset was generated with the peat from three LUs (i.e. PF, DF and OP) located on Sumatra's deep peat coastal plain in the Indonesian province of Jambi. The PF was less than 60 km from the other two LUs and the DF and OP were approximately 2 km apart (Fig. 1). The climate in the region is humid tropical. Long-term records from the nearest permanent weather station indicated that the average annual rainfall is 2466 mm y^–1^, and the mean minimum and maximum monthly temperatures are 22.7°C and 32.7°C, respectively [6].

### 2.2 Incubation method

For the incubation experiment, a total of 360 intact soil cores were collected (i.e. 120 per LU). At the three sampling sites, the intact cores were collected at random positions in a radius of 50 m. The soil cores had a volume of 313 cm^3^ (inner diameter 8.15 cm, height 6 cm) and were collected using a stainless steel core soil sampler from the upper part of the soil profile (0–6 cm). The soil cores were kept in their stainless steel containers with impermeable bottom lid at 4°C until moisture adjustment. To create a moisture gradient with the cores while minimally disturbing them the following handlings were applied randomly to the cores: oven dried at 70°C for 5 days; air dried for one to five days; extra 10 to 40 ml of distilled water application. The outcome was a continuum of different WFPS values between 0 and 95% (Appendix A). After the moisturizing handlings were completed, each individual intact soil core was placed into a air-tight 2.4 dm^3^ plastic container. The incubation took place in the lab simulating field temperature regime (i.e. 27°C ±2). The CO_2_ concentration in the containers was assessed at 0, 24, 48 and 72 hours by connecting the containers in closed system with an infrared gas analyzer (IRGA). The CO_2_ flux was calculated with a linear regression for the four time points. Gas fluxes (mg gas g dry soil^−1^ day^−1^) were calculated using Eq. (1):

(1) $$\text{Flux}=\frac{\delta \text{gas}\left(\frac{\mu \text{mol}}{\text{mol}}\right)}{\delta t}\times \frac{\text{Chamber}\;\text{volume}\;\left(L\right)\times \text{Mole}\;\text{of}\;\text{gas}\;\left(\frac{\text{mol}}{L}\right)\times \text{Molecular}\;\text{weight}\;\text{of}\;\text{gas}\;\left(\frac{g}{\text{mol}}\right)}{\text{Weight}\;\text{of}\;\text{dry}\;\text{peat}\;\text{soil}\;(g)}$$

The incubated cores that produced a lineal regression R^2^ below 0.98 were rejected and are marked as “ns” in Appendix A. Accordingly, for the PF, DF and OP, 14, 19 and 10 incubated cores were rejected, respectively. Because the containers remained sealed during the entire incubation period no moisture was lost and the bulk density and WFPS of each individual core was assessed after the completion of the incubation following Gregorich method [7].

### 2.3 Peat chemical and physical properties analyses

The soil was classified according to the World Reference Base [3]. In addition to the intact cores used for the incubation, extra samples were collected for physical and chemical analysis. Peat samples were fractionated into very light fraction plus light fraction (VLF, density less than 1 g ml^−1^), light (LF, density less than 1.25 g ml^−1^), medium weight fraction (MWF, density between 1.25 and 1.7 g ml^−1^) and heavy fraction (HF, density more than 1.7 g ml^−1^) with a dense liquid (NaI) following Gregorich method [5] (Table 2, Appendix B). The fractionated materials were dried, finely ground with a ball mill, and subsequently analyzed for total C and N content using a Costech Elemental Combustion System (Costech Analytical 191 Technologies, Inc.) coupled to a Delta V Advantage Mass Spectrometer (Thermo Fisher 192 Scientific Inc.). Soil pH (H_2_O 1:4) was determined according to van Reeuwijk method [8] and bulk density using soil cores (inner diameter 8.15 cm, height 6 cm following Gregorich method [7] (Table 3, Appendix C). The WFPS (ratio in percentage of volumetric soil water content to total soil porosity) was assessed following Anderson et al. [9] and was calculated using Eq. (2):

(2) $$WFPS=\left(\Theta_{m}\times \rho_{B}\right)/\left(1-\rho_{B}/\rho_{P}\right)$$

where Θm is gravimetric water content (g/g), ρB is bulk density (g/cm^3^), and ρP is particle density (2.65 g/cm^3^). The bulk density and gas samples were numbered in order they were assessed (i.e. #1 first and 360 last).

## CRediT authorship contribution statement

**Louis-Pierre Comeau:** Conceptualization, Methodology, Writing – original draft. **Kristell Hergoualc'h:** Visualization, Funding acquisition, Writing – review & editing. **Louis V. Verchot:** Funding acquisition, Writing – review & editing.

## Declaration of Competing Interest

The authors declare that they have no known competing financial interests or personal relationships which have, or could be perceived to have, influenced the work reported in this article.

## Figures and Tables

**Table:** 

Land-use^a^	sample #	CO_2_ flux (µg CO_2_-C g^−1^ d.w. h^−1^)^b^	WFPS^c^ (%)
PF	1	0.36	3.12
PF	2	0.22	2.45
PF	3	0.20	2.81
PF	4	ns	5.51
PF	5	0.17	4.65
PF	6	0.04	0.00
PF	7	0.07	0.32
PF	8	0.04	0.10
PF	9	0.09	0.00
PF	10	0.27	2.17
PF	11	0.08	0.03
PF	12	0.10	0.00
PF	13	0.09	0.00
PF	14	0.12	1.03
PF	15	0.08	0.00
PF	16	0.08	0.00
PF	17	0.09	1.01
PF	18	0.20	1.74
PF	19	0.06	0.00
PF	20	ns	0.00
PF	21	3.51	67.35
PF	22	4.36	67.32
PF	23	5.98	87.59
PF	24	15.41	62.85
PF	25	4.65	65.92
PF	26	7.37	67.96
PF	27	12.61	56.67
PF	28	2.98	62.73
PF	29	15.59	62.86
PF	30	20.52	61.57
PF	31	15.79	76.17
PF	32	ns	19.49
PF	33	7.92	73.55
PF	34	ns	71.30
PF	35	6.46	73.23
PF	36	14.76	71.56
PF	37	23.18	73.32
PF	38	2.32	72.46
PF	40	4.08	73.42
PF	41	5.42	71.81
PF	42	5.18	71.82
PF	43	4.55	76.72
PF	44	9.79	58.43
PF	45	4.56	68.47
PF	46	9.68	50.76
PF	47	12.39	72.92
PF	48	3.97	76.17
PF	49	2.65	78.03
PF	50	70.58	26.80
PF	51	9.24	41.86
PF	52	3.38	74.10
PF	53	2.38	65.13
PF	54	2.73	65.65
PF	55	4.17	81.74
PF	56	4.39	84.36
PF	57	2.70	82.75
PF	58	2.92	75.24
PF	59	3.38	78.27
PF	60	3.16	80.34
PF	61	1.35	82.78
PF	62	2.31	83.59
PF	63	ns	79.25
PF	64	2.87	83.21
PF	65	1.69	79.54
PF	66	ns	64.39
PF	67	1.83	93.84
PF	68	3.41	81.98
PF	69	9.91	45.49
PF	70	1.89	75.76
PF	71	ns	51.06
PF	72	1.39	86.10
PF	73	2.08	81.41
PF	74	1.46	74.78
PF	75	ns	78.23
PF	76	ns	89.85
PF	77	2.03	82.21
PF	78	1.78	80.06
PF	79	5.18	82.05
PF	80	ns	79.09
PF	81	3.88	74.20
PF	82	1.95	0.00
PF	83	9.31	62.23
PF	84	ns	80.26
PF	85	3.24	81.90
PF	86	4.30	75.95
PF	87	2.77	77.91
PF	88	2.74	90.69
PF	89	1.45	77.34
PF	90	ns	71.69
PF	91	3.90	69.84
PF	92	46.60	33.91
PF	93	4.23	66.39
PF	94	3.07	76.75
PF	95	3.19	82.84
PF	96	1.88	79.00
PF	97	2.72	85.06
PF	98	3.38	78.92
PF	99	1.61	80.50
PF	100	2.44	83.69
PF	101	12.59	54.87
PF	102	7.51	69.53
PF	103	2.97	62.00
PF	104	3.19	79.37
PF	105	1.91	75.49
PF	106	2.63	81.50
PF	107	2.51	75.01
PF	108	1.35	73.02
PF	109	36.29	76.32
PF	110	1.00	74.29
PF	111	1.39	83.07
PF	112	1.38	84.23
PF	113	2.47	69.07
PF	114	1.01	88.77
PF	115	2.66	85.94
PF	116	5.93	82.78
PF	117	1.02	79.68
PF	119	1.56	83.70
PF	120	1.74	80.70
DF	121	0.04	0.00
DF	122	0.07	0.00
DF	123	ns	0.25
DF	124	0.07	0.27
DF	125	0.09	0.35
DF	126	0.05	0.38
DF	127	0.06	0.41
DF	128	ns	0.44
DF	129	0.07	0.45
DF	130	0.05	0.57
DF	131	0.08	0.59
DF	132	0.05	0.61
DF	133	0.09	0.78
DF	134	0.07	0.87
DF	135	0.06	1.02
DF	136	0.08	1.35
DF	137	0.11	1.75
DF	138	0.13	4.22
DF	139	0.15	5.32
DF	140	ns	5.59
DF	141	4.79	29.02
DF	142	5.52	34.47
DF	143	5.02	35.41
DF	144	6.17	35.84
DF	145	11.69	41.63
DF	146	10.42	42.15
DF	147	2.62	43.05
DF	148	2.71	43.22
DF	149	ns	45.51
DF	150	11.04	46.66
DF	151	19.26	47.01
DF	152	3.64	47.98
DF	153	5.47	49.03
DF	154	13.74	50.03
DF	155	5.07	51.26
DF	156	5.90	51.43
DF	157	6.54	52.81
DF	158	ns	53.12
DF	159	ns	54.52
DF	160	7.67	57.41
DF	161	4.86	57.43
DF	162	ns	59.39
DF	163	3.05	59.54
DF	164	5.08	60.98
DF	165	ns	60.98
DF	166	6.51	61.55
DF	167	7.05	61.86
DF	168	1.66	62.30
DF	169	5.71	62.82
DF	170	4.84	64.20
DF	171	ns	64.70
DF	172	5.49	64.89
DF	173	2.01	65.14
DF	174	2.56	66.71
DF	175	1.79	68.37
DF	176	ns	68.48
DF	177	2.59	68.67
DF	178	6.76	68.85
DF	179	17.58	69.16
DF	180	4.52	70.16
DF	181	4.08	70.43
DF	182	5.66	70.91
DF	183	2.94	71.13
DF	184	5.04	71.16
DF	185	7.70	71.96
DF	186	5.27	72.02
DF	187	3.35	72.21
DF	188	6.24	72.22
DF	189	1.87	72.32
DF	190	5.31	72.61
DF	191	3.45	73.07
DF	192	2.37	73.10
DF	193	3.82	73.50
DF	194	2.04	73.59
DF	195	11.11	73.94
DF	196	ns	74.30
DF	197	4.50	74.44
DF	198	4.59	74.53
DF	199	10.91	74.81
DF	200	ns	75.28
DF	201	3.63	75.34
DF	202	5.22	75.84
DF	203	4.96	76.09
DF	204	2.85	76.36
DF	205	2.30	76.49
DF	206	2.27	76.68
DF	207	3.10	76.91
DF	208	ns	77.45
DF	209	4.68	77.46
DF	210	2.56	77.52
DF	211	3.95	77.89
DF	212	2.70	77.96
DF	213	2.82	78.03
DF	214	ns	78.27
DF	215	2.44	78.71
DF	216	2.81	78.88
DF	217	3.59	78.99
DF	218	ns	79.64
DF	219	2.22	79.81
DF	220	3.10	79.90
DF	221	ns	80.03
DF	222	4.76	80.21
DF	223	3.16	80.25
DF	224	ns	80.61
DF	225	ns	80.70
DF	226	4.07	80.73
DF	227	2.46	80.77
DF	228	7.05	81.53
DF	229	2.99	81.59
DF	230	4.97	81.77
DF	231	2.09	82.29
DF	232	ns	82.66
DF	233	2.49	83.42
DF	234	5.56	83.43
DF	235	5.13	84.39
DF	236	4.04	84.79
DF	237	2.37	84.83
DF	238	2.73	85.61
DF	239	5.76	85.91
DF	240	3.68	86.95
OP	241	0.05	0.00
OP	242	0.03	0.00
OP	243	ns	0.00
OP	244	0.03	0.00
OP	245	0.04	0.01
OP	246	ns	0.01
OP	247	0.04	0.03
OP	248	0.05	0.04
OP	249	0.04	0.05
OP	250	0.03	0.09
OP	251	0.02	0.13
OP	252	0.02	0.13
OP	253	0.04	0.17
OP	254	0.03	0.43
OP	255	0.02	0.55
OP	256	0.03	0.78
OP	257	0.03	0.90
OP	258	0.09	2.65
OP	259	0.18	4.89
OP	260	0.18	6.42
OP	261	2.53	17.45
OP	262	4.27	18.33
OP	263	2.25	22.77
OP	264	2.88	23.86
OP	265	1.21	26.10
OP	266	2.41	26.92
OP	267	1.62	27.73
OP	268	8.64	27.79
OP	269	3.04	27.92
OP	270	2.57	28.38
OP	271	4.14	28.91
OP	272	1.49	30.70
OP	273	0.96	31.53
OP	274	1.23	32.18
OP	275	2.93	33.15
OP	276	ns	33.71
OP	277	6.17	34.64
OP	278	0.74	35.79
OP	279	ns	39.73
OP	280	1.82	40.66
OP	281	1.41	40.70
OP	282	2.23	41.58
OP	283	1.72	43.80
OP	284	6.66	43.89
OP	285	2.41	45.33
OP	286	0.70	45.69
OP	287	1.49	47.09
OP	288	0.91	47.13
OP	289	ns	47.51
OP	290	3.75	47.65
OP	291	0.78	49.06
OP	292	1.18	49.90
OP	293	0.50	50.18
OP	294	1.05	50.82
OP	295	1.10	51.03
OP	296	2.96	52.23
OP	297	1.54	52.26
OP	298	0.04	53.58
OP	299	1.35	53.88
OP	300	1.08	53.93
OP	301	0.96	54.12
OP	302	1.28	54.33
OP	303	1.15	54.48
OP	304	1.37	54.68
OP	305	2.01	55.35
OP	306	2.70	55.37
OP	307	0.72	55.73
OP	308	1.60	56.23
OP	309	ns	56.57
OP	310	1.55	57.54
OP	311	1.79	57.69
OP	312	1.48	57.83
OP	313	1.04	58.94
OP	314	1.82	58.98
OP	315	1.34	59.22
OP	316	1.19	59.40
OP	317	1.44	60.94
OP	318	1.75	61.92
OP	319	1.33	62.28
OP	320	ns	62.59
OP	321	ns	62.98
OP	322	2.35	63.45
OP	323	1.44	63.55
OP	324	1.34	64.04
OP	325	1.88	64.05
OP	326	1.38	64.20
OP	327	2.32	64.24
OP	328	2.40	64.35
OP	329	1.08	64.60
OP	330	3.45	65.00
OP	331	2.93	66.19
OP	332	2.47	66.65
OP	333	1.43	66.93
OP	334	1.95	66.96
OP	335	1.33	67.00
OP	336	0.62	67.86
OP	337	1.26	67.95
OP	338	0.81	68.27
OP	339	1.25	68.44
OP	340	1.40	69.26
OP	341	1.76	69.98
OP	342	1.23	70.06
OP	343	ns	70.46
OP	344	0.69	70.65
OP	345	1.42	70.83
OP	346	1.16	72.08
OP	347	3.14	72.42
OP	348	1.71	72.49
OP	349	0.84	73.05
OP	350	1.51	73.36
OP	351	1.33	74.24
OP	352	ns	74.98
OP	353	2.06	75.18
OP	354	1.07	77.10
OP	355	0.73	77.49
OP	356	1.56	77.57
OP	357	2.15	77.79
OP	358	2.82	79.14
OP	359	0.47	82.24
OP	360	0.82	82.50

a PF, primary peat swamp forest; DF, drained and logged forest on peat soil; 7 year old oil palm plantation on peat soil.

b d.w., dry weight.

C WFPS, water filled pore space.

**Table:** 

Land-use^a^	Soil profile	replicate	Bulk density (g d.w. cm^−3^)^b^	Soil pH (H_2_O 1:4)
PF	A	1	0.16	3.14
PF	A	2	0.12	3.13
PF	A	3	0.15	3.14
PF	A	4	0.18	3.15
PF	B	1	0.16	3.05
PF	B	2	0.16	3.80
PF	B	3	0.14	3.14
PF	B	4	0.14	3.32
PF	C	1	0.15	3.18
PF	C	2	0.18	3.18
PF	C	3	0.14	3.20
PF	C	4	0.15	3.17
DF	D	1	0.18	3.82
DF	D	2	0.13	3.46
DF	D	3	0.18	3.26
DF	D	4	0.16	3.37
DF	E	1	0.13	3.11
DF	E	2	0.16	2.93
DF	E	3	0.18	3.11
DF	E	4	0.17	3.56
DF	F	1	0.19	3.58
DF	F	2	0.15	3.21
DF	F	3	0.13	3.21
DF	F	4	0.14	3.21
OP	G	1	0.16	3.52
OP	G	2	0.18	4.14
OP	G	3	0.20	3.42
OP	G	4	0.17	3.83
OP	H	1	0.29	3.47
OP	H	2	0.21	4.76
OP	H	3	0.17	3.21
OP	H	4	0.19	3.44
OP	I	1	0.20	3.54
OP	I	2	0.20	3.06
OP	I	3	0.21	3.06
OP	I	4	0.20	3.06

a PF, intact peat swamp forest; DF, drained logged forest; OP, 7-year-old oil palm plantation.

b d.w., dry weight.

**Table:** 

Land-use^a^	Soil profile	Soil fraction^b^	Weight Proportion of the fraction (%)	Carbon (%)	Nitrogen (%)	C:N (ratio)
PF	A	VLF	0.1%	73.1	1.5	50.0
PF	A	LF	5.4%	79.1	1.1	72.5
PF	A	MF	92.1%	52.9	1.5	36.2
PF	A	HF	2.4%	47.0	1.1	43.1
PF	B	VLF	0.1%	55.6	0.7	83.9
PF	B	LF	1.1%	57.8	1.1	51.7
PF	B	MF	77.4%	47.8	1.4	33.6
PF	B	HF	21.4%	52.0	1.5	35.1
PF	C	VLF	0.1%	57.3	1.4	40.8
PF	C	LF	51.4%	56.2	1.5	38.3
PF	C	MF	29.4%	55.0	1.5	37.7
PF	C	HF	19.2%	53.6	1.1	47.9
DF	D	VLF	0.8%	56.1	1.1	50.8
DF	D	LF	6.2%	56.9	1.2	49.0
DF	D	MF	90.3%	49.1	1.3	38.2
DF	D	HF	2.7%	50.4	1.3	40.3
DF	E	VLF	2.1%	52.1	1.6	33.4
DF	E	LF	16.2%	51.4	1.7	30.8
DF	E	MF	12.7%	44.6	1.6	28.3
DF	E	HF	69.0%	49.3	1.8	28.1
DF	F	VLF	0.1%	53.6	2.2	24.8
DF	F	LF	3.1%	55.6	1.3	42.1
DF	F	MF	5.0%	53.4	1.5	35.5
DF	F	HF	91.8%	54.0	1.6	33.9
OP	G	VLF	0.1%	53.6	0.9	61.6
OP	G	LF	1.8%	55.4	1.2	46.2
OP	G	MF	14.5%	50.8	1.4	37.3
OP	G	HF	83.6%	52.4	1.3	41.3
OP	H	VLF	1.9%	55.1	1.2	44.4
OP	H	LF	44.3%	55.4	1.4	38.4
OP	H	MF	25.5%	51.0	1.4	36.4
OP	H	HF	28.3%	51.5	1.4	37.2
OP	I	VLF	0.3%	56.7	1.4	41.8
OP	I	LF	7.7%	56.3	1.4	40.8
OP	I	MF	5.9%	53.7	1.3	40.3
OP	I	HF	86.0%	53.4	1.4	37.5

a PF, intact peat swamp forest; DF, drained logged forest; OP, 7-year-old oil palm plantation.

b VLF, very light fraction; LF, light fraction; MWF, medium weight fraction; HF, heavy fraction.

## References

[1] Comeau L.P., Lai D.Y.F., Cui J.J., Hartill J. (2018).

[2] Comeau L.P. (July 2016).

[3] IUSS Working Group WRB, (2006).

[4] Hergoualc'h K., Verchot L.V. (2011). Stocks and fluxes of carbon associated with land use change in Southeast Asian tropical peatlands: a review. Glob. Biogeochem. Cycles.

[5] Hergoualc'h K.A., Verchot L.V. (2012). Changes in soil CH4 fluxes from the conversion of tropical peat swamp forests: a meta-analysis. J. Integr. Environ. Sci..

[6] Siderius C. (2004).

[7] Gregorich E.G., Beare M.H., Mckim U.F., Skjemstad J.O. (2006). Chemical and biological characteristics of physically uncomplexed organic matter. Soil Sci. Soc. Am. J..

[8] van Reeuwijk L.P. (2002). Procedures for soil analysis: Wageningen, International Soil Reference and Information Centre.

[9] Anderson F.L., Cooper J.A., Amador J.A. (2019). Laboratory-scale evaluation of the effects of water-filled pore space on emissions of CO_2_, CH_4_, N_2_O, and N_2_ from soil based wastewater treatment. Water Air Soil Pollut.

